# Ringing phenomenon based measurement of weak mode-coupling strength in an optical microresonator

**DOI:** 10.1038/s41598-017-16961-7

**Published:** 2017-12-12

**Authors:** Ming-Yong Ye, Mei-Xia Shen, Xiu-Min Lin

**Affiliations:** 1 Fujian Provincial Key Laboratory of Quantum Manipulation and New Energy Materials, College of Physics and Energy, Fujian Normal University, Fuzhou, 350117 China; 2 Fujian Provincial Collaborative Innovation Center for Optoelectronic Semiconductors and Efficient Devices, Xiamen, 361005 China; 3 Key Laboratory of Quantum Information, University of Science and Technology of China, Chinese Academy of Sciences, Hefei, 230026 China

## Abstract

There is always a coupling between the degenerate clockwise (CW) and counter-clockwise (CCW) modes in a whisperinggallery- mode (WGM) optical microresonator, since the surface of the microresonator can not be perfectly smooth. It is important to measure this coupling strength in many applications. When the coupling strength is strong, the conventional method by observing mode splitting in the stationary spectrum can be used to measure its value. However, when the coupling strength is weak, the conventional method will not work. We experimentally demonstrate that the ringing phenomenon can be used to measure weak coupling strength between the CW and CCW modes in a WGM optical microresonator.

## Introduction

When there is a coupling between two degenerate modes in a quantum or an optical system, the two degenerate modes will be combined to form two new modes with different energies or frequencies. Whispering-gallery-mode (WGM) optical microresonators such as microsphere, microdisk and microtoroid receive great interests in recent years due to their high quality (Q) factors and small mode volumes^1^. They provide a good research platform in highly sensitive sensing^2,3^, optomechanics^4,5^, lasing^6,7^, and so on^8–15^. The WGM microresonators can support degenerate clockwise (CW) and counter-clockwise (CCW) modes. Their rough surfaces can induce Rayleigh scattering and lead to a coupling between the degenerate CW and CCW modes. Experimental observation of the mode coupling phenomenon in WGM microresonators has been reported by many researchers^16–19^. In a recent progress, spontaneous emergence of chirality in WGM microresonators has been experimentally demonstrated, where there is a Kerr-nonlinearity-modulated coupling between the CW and CCW modes^20,21^.

The stationary transmission spectrum of a high-Q WGM resonance can be obtained by using a continuous laser to sweep the resonance in a slow speed. In the stationary transmission spectrum, the strong coupling between the degenerate CW and CCW modes is manifested as a single transmission dip splitting into a doublet^16,22^. The strong coupling strength can be calculated by measuring the splitting. However, this conventional method has a limit that it is not applicable for measuring weak coupling strength, because in the method the coupling strength should be large enough so that the doublet can be resolved. The ability to measure weak coupling strength between the CW and CCW modes is not only a scientific pursuit but also has some practical applications. Zhu *et al*. experimentally showed that the deposition of nanoparticles on the WGM microresonator could be detected by observing the change of the coupling strength between the CW and CCW modes^22^. The ability of measuring smaller coupling strength means the ability of detecting smaller particles.

When a continuous laser is used to sweep over a high-Q WGM resonance in a fast speed, the ringing phenomenon will be observed^23–25^. It results from an interference between the decaying light from the WGM microresonator and the directly transmitted light in the coupling waveguide. The ringing phenomenon has been observed in many WGM optical microresonators^24–32^. When there is a strong coupling between the CW and CCW modes in the WGM microresonator, the coupling strength can be measured from the ringing phenomenon^27^. It is not clear whether the ringing phenomenon method is also applicable when the coupling strength is weak. Here we give an experimental demonstration that the weak coupling strength between the CW and CCW modes can be measured from the ringing phenomenon.

## Results

### Theoretical background

**Figure 1:**
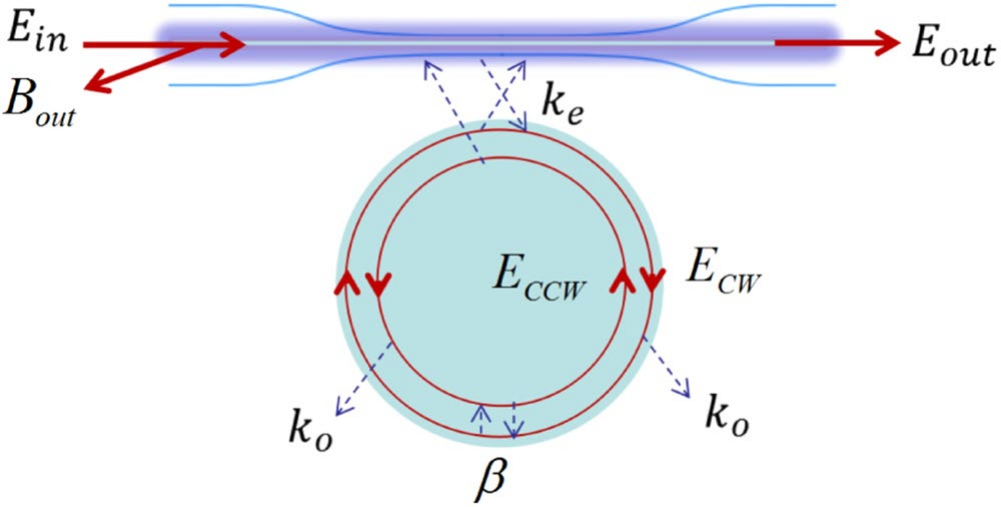
Schematic illustration of a WGM microresonator coupled to a fiber taper. The degenerate CW and CCW modes in the microresonator have a coupling, so that the input light *E*_*in*_ can lead to both transmission *E*_*out*_ and reflection *B*_*out*_.

We first give some theoretical background on our experiment. Suppose a fiber taper is used as a waveguide to excite a WGM microresonator. A sketch of the experimental configuration is presented in Fig. 1. The CW and CCW modes in the microresonator are denoted by *E*_*cw*_ and *E*_*ccw*_, respectively. The input laser is denoted by *E*_*in*_(*t*), which is used to excite the CW mode. The output from the fiber taper is denoted by *E*_*out*_ and the reflection is denoted by *B*_*out*_. The CW mode has a coupling with the CCW mode, and their evolutions satisfy the following equations^17,22^

1 $$\frac{d{E}_{cw}(t)}{dt}=k{E}_{cw}(t)+j\beta {E}_{ccw}(t)+\sqrt{2{k}_{e}}{E}_{in}(t),$$

2 $$\frac{d{E}_{ccw}(t)}{dt}=k{E}_{ccw}(t)+j\beta {E}_{cw}(t),$$

with $k=-j({w}_{c}-\beta )-{k}_{o}-{k}_{e}$, where *w*_*c*_ is the resonance angular frequency of the degenerate CW and CCW modes, *k*_*o*_ denotes the intrinsic loss rate, *k*_*e*_ represents the loss rate associated with the coupling to the fiber taper, and *β* (*β* > 0) is the coupling strength between the CW and CCW modes. In the above we have neglected the loss of the CW and CCW modes due to the modal coupling^17^. Assume that the instantaneous angular frequency of the input laser is decreasing in a constant speed *v*, the input can be described as

3 $${E}_{in}(t)=s{e}^{-j({w}_{{l}_{0}}t-\frac{1}{2}v{t}^{2})},\,v > \mathrm{0,}$$

where *s* represents the amplitude of the input laser, and ${w}_{{l}_{0}}$ is the angular frequency of the input laser at *t* = 0. Derivative the phase of *E*_*in*_(*t*) with *t*, it can be found that the instantaneous angular frequency of the input is ${w}_{l}(t)={w}_{{l}_{0}}-vt$. Suppose at the initial time *t* = 0, there is *E*_*cw*_ = *E*_*ccw*_ = 0 and the initial angular frequency of the input laser is much larger than the resonance angular frequency of the mode, i.e., ${w}_{{l}_{0}} >  > {w}_{c}$, the angular frequency of the laser will reduce from *t* = 0 and sweep over the coupled modes in the constant speed *v*. Define

4 $${E}_{1}(t)={E}_{cw}(t)+{E}_{ccw}(t),$$

5 $${E}_{2}(t)={E}_{cw}(t)-{E}_{ccw}(t\mathrm{).}$$

We have two independent equations

6 $$\frac{d{E}_{1}(t)}{dt}={k}_{1}{E}_{1}(t)+\sqrt{2{k}_{e}}{E}_{in}(t),$$

7 $$\frac{d{E}_{2}(t)}{dt}={k}_{2}{E}_{2}(t)+\sqrt{2{k}_{e}}{E}_{in}(t),$$

where ${k}_{1}=-j({w}_{c}-2\beta )-{k}_{o}-{k}_{e}$ and ${k}_{2}=-j{w}_{c}-{k}_{o}-{k}_{e}$. The output is linked to the input and the field in the microresonator through

8 $${E}_{out}(t)=-{E}_{in}(t)+\sqrt{2{k}_{e}}{E}_{cw}(t)=-{E}_{in}(t)+\sqrt{2{k}_{e}}({E}_{1}(t)+{E}_{2}(t))/\mathrm{2,}$$

and the normalized transmission of the fiber taper in the time domain is

9 $$T(t)=|{E}_{out}(t)/{E}_{in}(t{)|}^{2}=|-1+\sqrt{2{k}_{e}}\{{E}_{1}(t)/{E}_{in}(t)+{E}_{2}(t)/{E}_{in}(t)\}/{\mathrm{2|}}^{2}\mathrm{.}$$

To understand qualitatively the shape of the normalized transmission, it is a good approximation in the undercoupling condition that

10 $$T(t)\approx \{{T}_{1}(t)+{T}_{2}(t)\}/2$$

with

11 $${T}_{1}(t)=|-1+\sqrt{2{k}_{e}}{E}_{1}(t)/{E}_{in}(t{)|}^{2},$$

12 $${T}_{2}(t)=|-1+\sqrt{2{k}_{e}}{E}_{2}(t)/{E}_{in}(t{)|}^{2}.$$

We denote the special normalized transmission *T*(*t*) with the modal coupling strength *β* = 0 as *T*_0_(*t*), it can be found that *T*_2_(*t*) has the same shape as *T*_0_(*t*) and *T*_1_(*t*) also has the same shape as *T*_0_(*t*) but there is a time shift Δ*t* = 2*β*/*v*. Therefore Eq. (10) shows that the normalized transmission *T*(*t*) with nonzero modal coupling strength *β* can be understood as an interference of two shifted *T*_0_(*t*), i.e.,

13 $$T(t)\approx \{{T}_{0}(t-{\rm{\Delta }}t)+{T}_{0}(t\mathrm{)\}/2,}\,{\rm{\Delta }}t=2\beta /v\mathrm{.}$$

The reflection amplitude has the expression

14 $${B}_{out}(t)=\sqrt{2{k}_{e}}{E}_{ccw}(t),$$

and the normalized reflection of the fiber taper in the time domain is

15 $$R(t)=|{B}_{out}(t)/{E}_{in}(t{)|}^{2}\mathrm{.}$$

**Figure 2:**
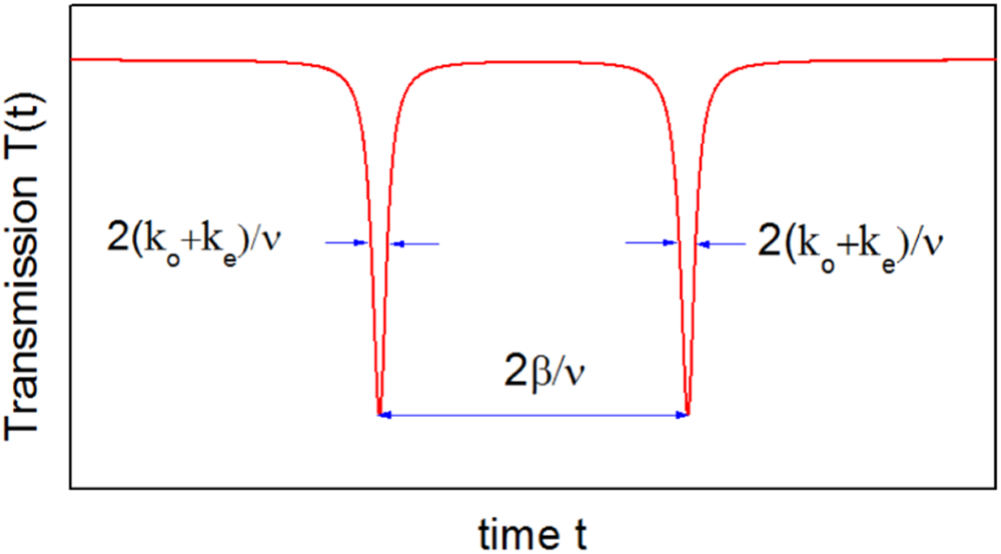
Schematic illustration of the stationary transmission spectrum of a WGM resonance. It can be obtained by using a continuous laser to sweep the resonance in a slow speed, where the CW and CCW modes has a large coupling. The condition *β* > *k*_*o*_ + *k*_*e*_ is needed to resolve the splitting.

The function shape of the transmission *T*(*t*) depends much on the laser sweeping speed *v*. The stationary transmission and reflection spectra of the WGM microresonator can be obtained when the laser sweeping speed *v* is much smaller than a characteristic speed *v*_0_^29^, which is defined as *v*_0_ = 4(*k*_*o*_ + *k*_*e*_)^2^. When the laser sweeping speed *v* is small and there is no mode coupling, i.e., *β* = 0, only the CW mode will be excited by the input laser and the stationary transmission *T*_0_(*t*) will be a dip of Lorentz shape with a width of 2(*k*_*o*_ + *k*_*e*_)/*v*. When the coupling strength *β* is not zero^16^, the CW and CCW modes will be coupled to form two new modes and both of them will be excited, then the stationary transmission dip will be split into a doublet with the time difference 2*β*/*v*, as shown in Fig. 2. The condition for the doublet to be resolved is that the splitting is larger than the width of the dip^22^, i.e. *β* > *k*_*o*_ + *k*_*e*_. Therefore when the coupling strength *β* is smaller than *k*_*o*_ + *k*_*e*_, it can not be measured from the stationary transmission spectrum by observing the splitting.

The ringing phenomenon in the transmission will be observed when the laser sweeping speed *v* is comparable or larger than the characteristic speed *v*_0_. When the laser sweeping speed *v* is large and the coupling strength *β* is zero, the normalized transmission *T*_0_(*t*) will be a standard ringing curve^23,30^. When the coupling strength *β* is not zero, the normalized transmission is an interference of two standard ringing curves as shown by Eq. (13). It can be found from Eqs (1) and (2) that the parameters *k*_*o*_, *k*_*e*_, *β* and *v* will determine the function shape of the transmission *T*(*t*). Conversely, using the theoretical transmission *T*(*t*) to fit the experimentally observed ringing curves, we can get the values of the parameters *k*_*o*_, *k*_*e*_, *β* and *v*. What we will show is that even when *β* < *k*_*o*_ + *k*_*e*_, the measurement of the coupling strength *β* can be achieved by fitting the experimentally observed ringing curves. This result extends the measurement range of the coupling strength *β* that is not possible before.

### Measurements

**Figure 3:**
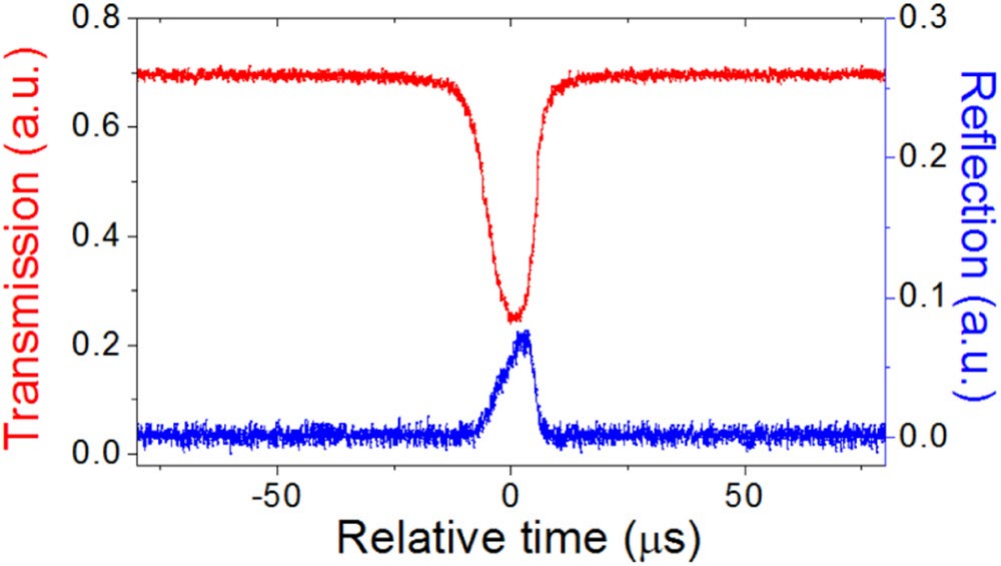
Experimental stationary transmission and reflection spectra of a resonance. The wavelength of the resonance is about 1554.75 nm. The laser sweeping speed *v*/2*π* is as slow as about 0.1 MHz/*μ*s.

**Figure 4:**
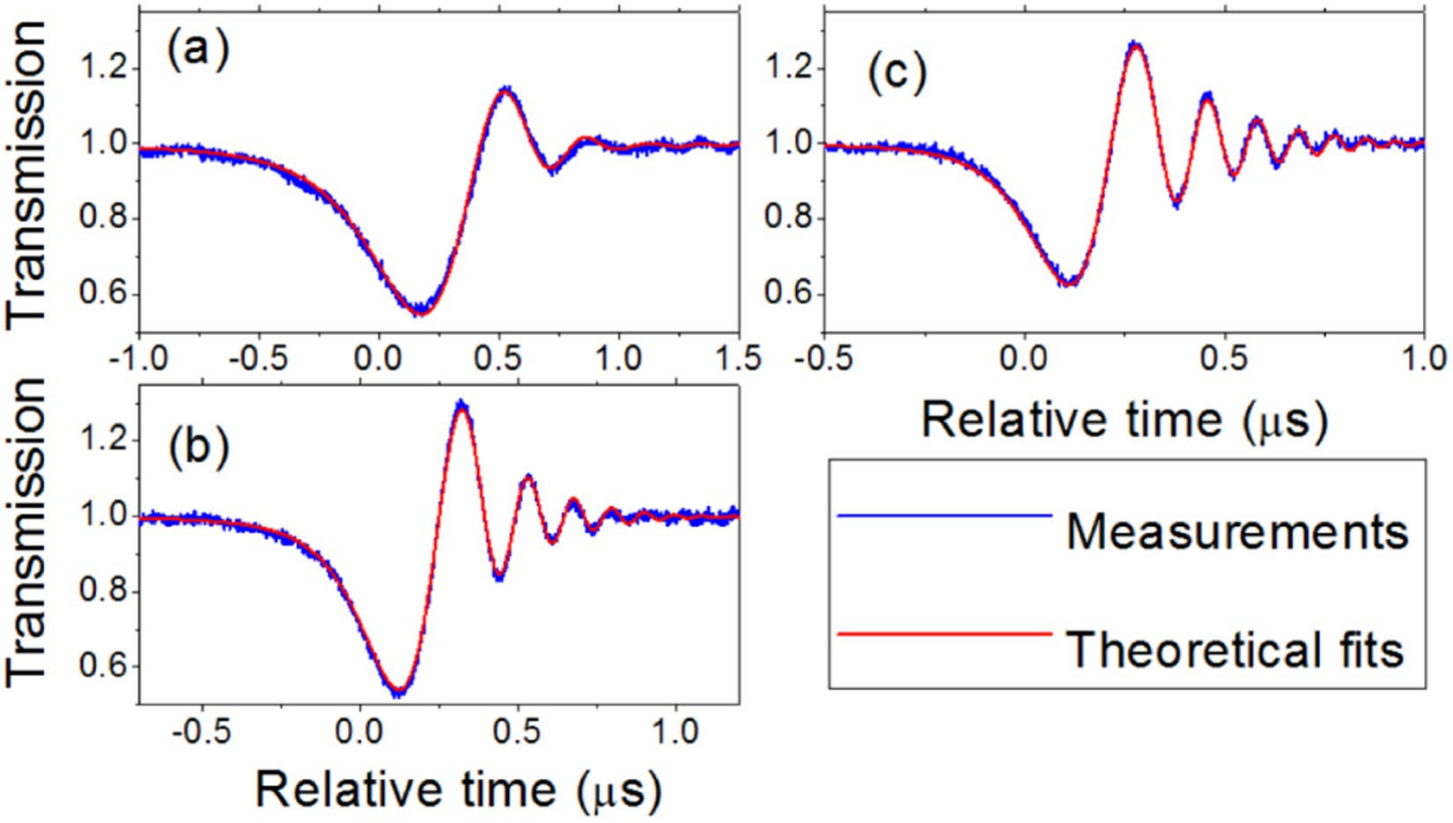
Experimental ringing curves in the transmission and their theoretical fits. The wavelength of the resonance is about 1554.75 nm. From (**a**–**c**) the laser sweeping speed is increased and the fitting parameters are shown in Table 1.

We used a fiber taper to couple light into and out of a silica microsphere (see the Methods), and studied a resonance with the wavelength about 1554.75 nm. Figure 3 shows its stationary transmission and reflection spectra, which was obtained when the laser sweeping speed was very slow. The existence of reflection shows that there is a coupling between the CW and CCW modes, while no mode splitting is observed in the transmission. Therefore it demonstrates a weak coupling strength between the CW and CCW modes that can not be measured by the conventional method through measuring the splitting. When the laser sweeping speed was increased, we observed the ringing phenomenon in the transmission. Figure 4 shows the ringing curves in the transmission with different fast laser sweeping speeds. It can be seen that a faster laser sweeping speed can lead to more oscillation. By fitting the experimental ringing curves we get the coupling strength *β*, the decay rates *k*_*o*_ and *k*_*e*_ that are displayed in Table 1. It can be found that although the ringing curves are obtained with different laser sweeping speed *v* and have different shapes, almost the same *β*, *k*_*o*_ and *k*_*e*_ are obtained. That the values of *k*_*e*_ in different curves are slightly different can be understood from the fact that the gap between the microsphere and the fiber taper were not strictly stable. Figure 4 shows that the weak coupling strength *β*, where *β* ≈ 0.4(*k*_*o*_ + *k*_*e*_), can be measured by observing the ringing phenomenon. We note that the intrinsic Q factor of the CW and CCW modes calculated from *w*_*c*_/(2*k*_*o*_) is about 2 × 10^8^ and the characteristic speed *v*_0_/2*π* ≈ 7.6 MHz/*μ*s.

**Table 1:** Parameters for theoretical fits in Fig. 4.

	a	b	c
*k*_*o*_/2*π* (MHz)	0.45	0.45	0.45
*k*_*e*_/2*π* (MHz)	0.10	0.13	0.11
*β*/2*π* (MHz)	0.22	0.22	0.22
*v*/2*π* (MHz/*μ*s)	4.5	11.5	15.5

**Figure 5:**
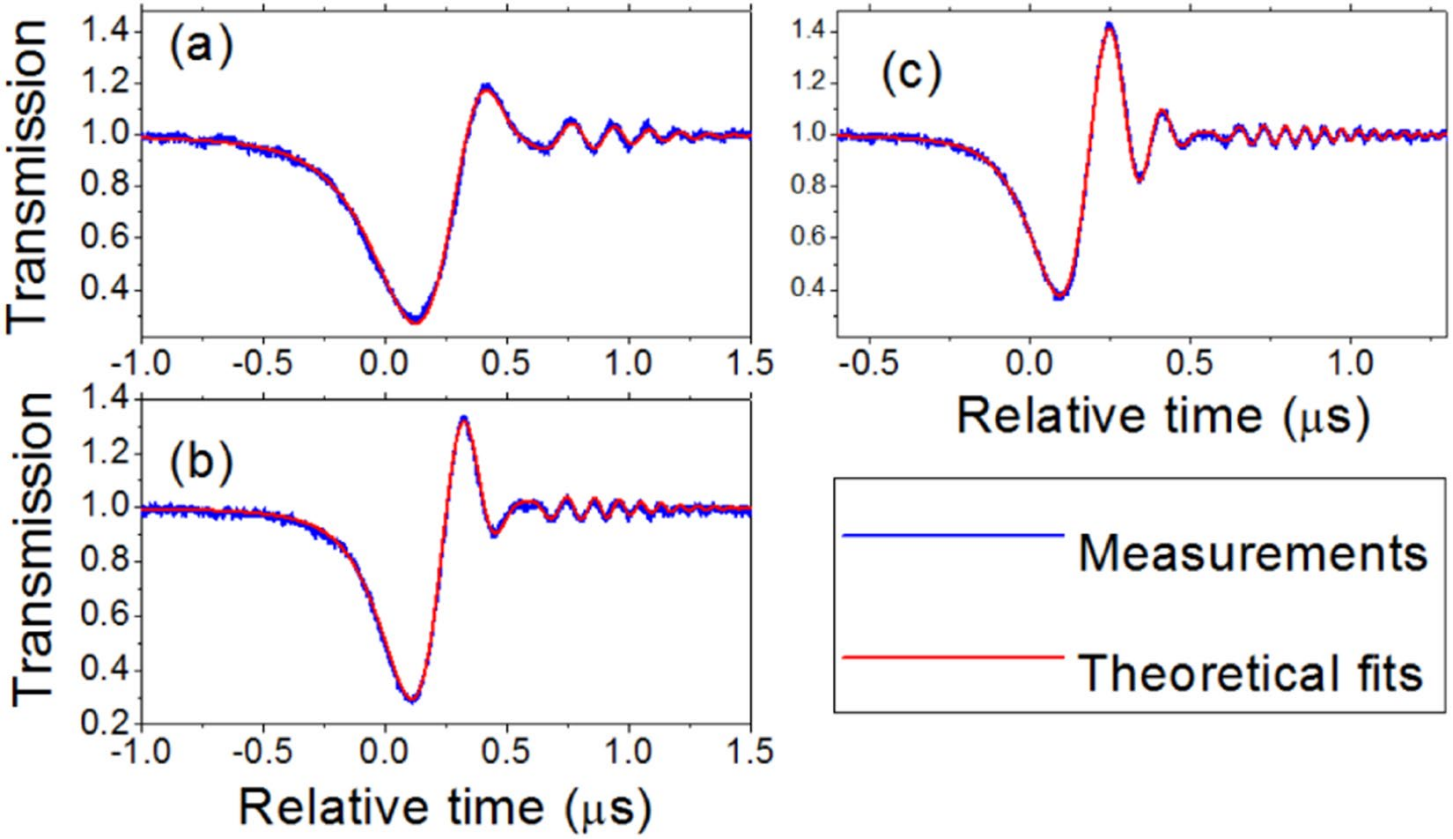
Experimental ringing curves in the transmission and their theoretical fits. The wavelength of the resonance is about 1533.29 nm. From (**a**–**c**) the laser sweeping speed is increased and the fitting parameters are shown in Table 2.

**Figure 6:**
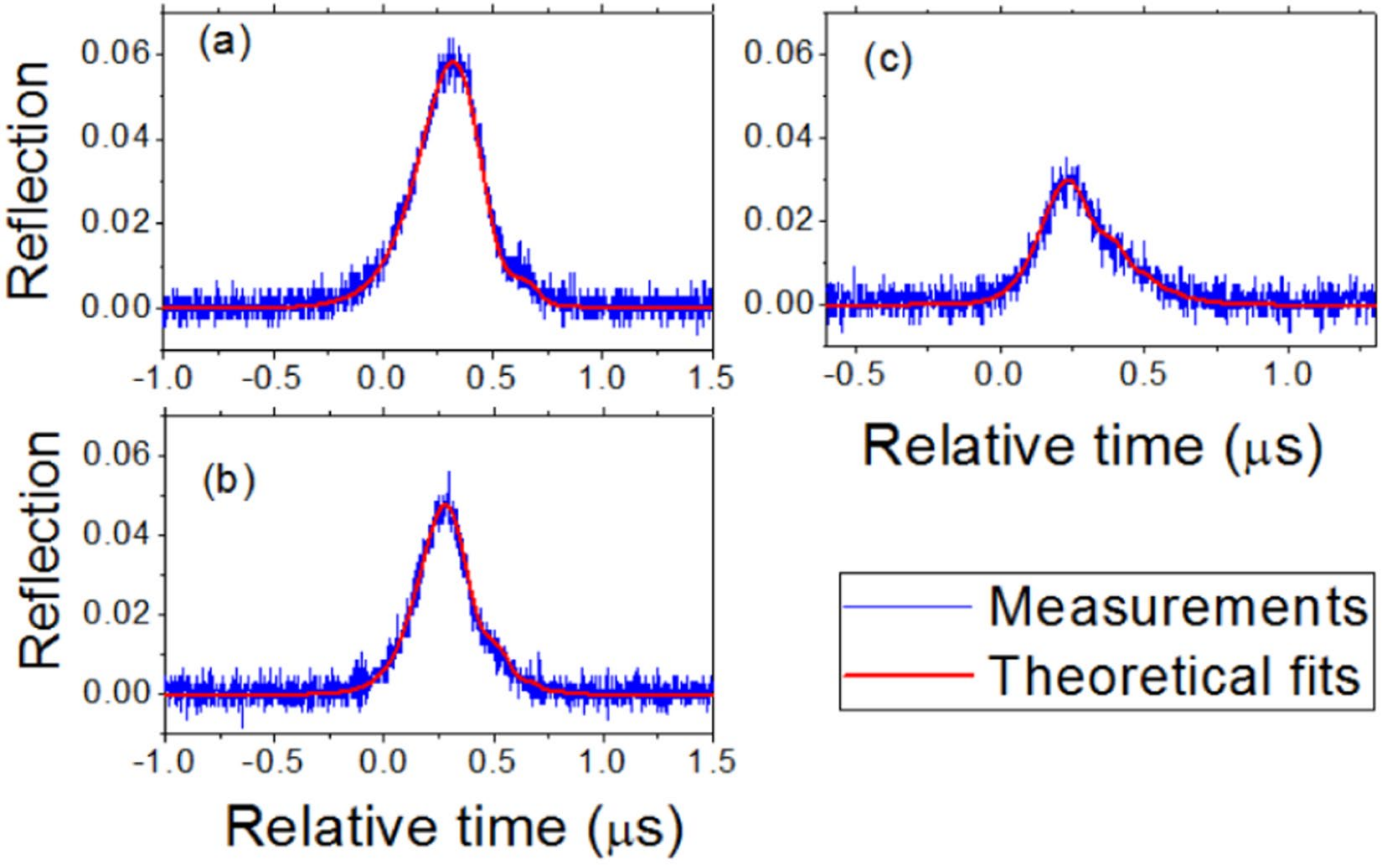
Experimental reflections and their theoretical fits with the fitting parameters shown in Table 2. The wavelength of the resonance is about 1533.29 nm.

Figure 5 exhibits the ringing curves from another resonance with the wavelength about 1533.29 nm, where the fitting parameters are given in Table 2. It is another example that the weak coupling strength *β* with *β* < *k*_*o*_ + *k*_*e*_ can not be measured by the conventional method but can be measured by the ringing phenomenon method. To confirm the correctness of the fitting parameters in Table 2, we also use these parameters to draw theoretical reflection curves according to Eq. (15). The theoretical reflection curves and experimental reflection curves are shown in Fig. 6. We note that each experimental reflection curve and its corresponding ringing curve in the transmission are recorded at the same time. It can be found that the theoretical reflection curves agree well with the experimental observations (see Fig. 6), which shows the correctness of the fitting parameters.

**Table 2:** Parameters for theoretical fits in Figs 5 and 6.

	a	b	c
*k*_*o*_/2*π* (MHz)	0.43	0.42	0.42
*k*_*e*_/2*π* (MHz)	0.24	0.25	0.24
*β*/2*π* (MHz)	0.43	0.44	0.46
*v/*2*π* (MHz/*μ*s)	7.0	11.1	19.3

## Summary

In summary, we have observed the ringing phenomenon in a silica microsphere, where there was a coupling between the CW and CCW modes. By fitting the observed ringing curves from a couple-mode theory, it is found that the theory agrees well with the measurement. The result shows that the weak coupling strength between the CW and CCW modes can be measured through observing the ringing phenomenon, which extends the measurement range of the mode-coupling strength in WGM microresonators.

## Methods

In the experiment, a fiber taper was used to couple light into and out of a silica microsphere. The microsphere was made by melting a single-mode fiber tip using a CO_2_ laser. The fiber taper was made by using a hydrogen flame to heat a single-mode fiber and simultaneously using a motorized translational stage to stretch the fiber from two sides. The fiber taper had a diameter of about 2 *μm*, and the microsphere had a diameter of about 100 *μm*, which was fixed on a nano-stage (Thorlabs MAX312D) so that we could control the gap between the microsphere and the fiber taper. A fiber coupler (10:90) was used as its function similar to a circulator. The 100% port of the fiber coupler was linked to the input side of the fiber taper. A wavelength tunable laser (Newport TLB-6728) was linked to the 10% port of the fiber coupler and a photoreceiver was linked to the 90% port of the fiber coupler, which detected the reflection light from the microsphere. The transmission light from the fiber taper was detected by another photoreceiver. The results from both photoreceivers were shown on an oscilloscope. The frequency sweeping of the laser was controlled by a triangle-wave signal from a function generator, which was also shown on the oscilloscope. In the experiment, the laser intensity was kept low to avoid thermal effect in the microsphere. The values of the fitting parameters can be obtained in a systematic way by using the least square method^27^, but as a demonstration we get them just by observing the difference between the theoretical and the experimental curves.

### Data Availability

The datasets generated during and/or analysed during the current study are available from the corresponding author on reasonable request.
