# Author Correction: Ringing phenomenon based measurement of weak mode-coupling strength in an optical microresonator

**DOI:** 10.1038/s41598-018-22999-y

**Published:** 2018-03-12

**Authors:** Ming-Yong Ye, Mei-Xia Shen, Xiu-Min Lin

**Affiliations:** 1 Fujian Provincial Key Laboratory of Quantum Manipulation and New Energy Materials, College of Physics and Energy, Fujian Normal University, Fuzhou, 350117 China; 2 Fujian Provincial Collaborative Innovation Center for Optoelectronic Semiconductors and Efficient Devices, Xiamen, 361005 China; 3 Key Laboratory of Quantum Information, University of Science and Technology of China, Chinese Academy of Sciences, Hefei, 230026 China

Correction to: *Scientific Reports* 10.1038/s41598-017-16961-7, published online 12 December 2017

The Acknowledgements section in this Article is incomplete.

“This work was supported by the National Natural Science Foundation of China (Grant Nos 11674059, 61275215), Fujian Provincial College Funds for Distinguished Young Scientists (Grant No. JA14070), Natural Science Foundation of Fujian Province (Grant Nos 2016J01008, 2016J01009), and Open Project of Key Laboratory of Quantum Information (Chinese Academy of Sciences) under Grant No. KQI201601.”

should read:

“This work was supported by the National Natural Science Foundation of China (Grant Nos. 11674059, 61275215), Fujian Provincial College Funds for Distinguished Young Scientists (Grant No. JA14070), Natural Science Foundation of Fujian Province (Grant Nos 2016J01008, 2016J01009), Open Project of Key Laboratory of Quantum Information (Chinese Academy of Sciences) under Grant No. KQI201601, and National Key R&D Program (Grant No. 2016YFA0301303).”

